# *Salmonella*—Host–microbiota interactions: Immunity and metabolism

**DOI:** 10.1002/imo2.16

**Published:** 2024-07-04

**Authors:** Bingxin Tang, Wenwen Cui, Xiao Li, Huan Yang

**Affiliations:** ^1^ Xuzhou Key Laboratory of Laboratory Diagnostics, School of Medical Technology Xuzhou Medical University Xuzhou China; ^2^ Xuzhou Center for Disease Control and Prevention Xuzhou China

## Abstract

*Salmonella* establish a foundation for systemic infection through induced inflammation and immune evasion. *Salmonella* manipulates host metabolism to favor its own proliferation within the host. *Salmonella* infection can disrupt the balance of gut commensal bacteria and use microbial metabolites to fuel its own energy metabolism.

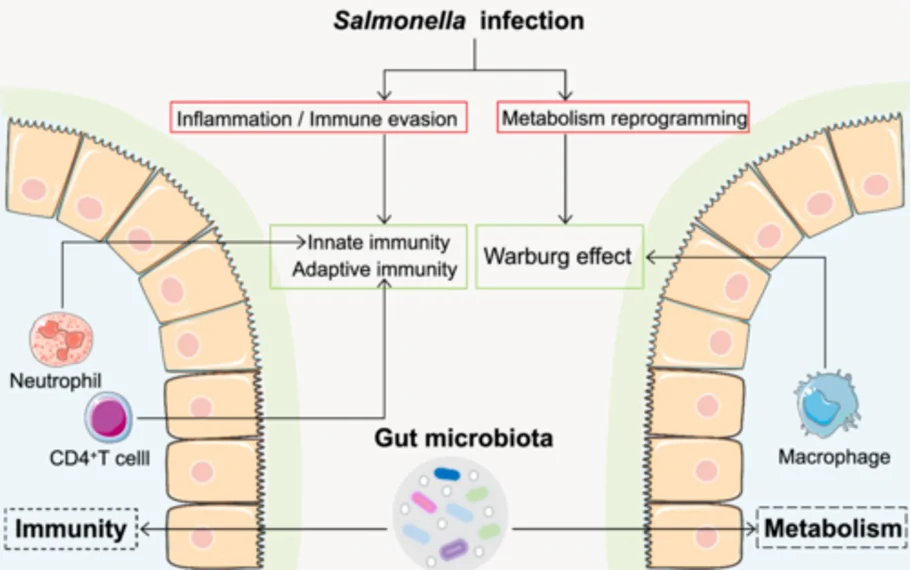

*Salmonella* is a group of zoonotic pathogens that causes intestinal infections, primarily transmitted through contaminated food or water. *Salmonella* invasion can trigger intestinal inflammation and exploit the electron receptors produced by inflamed cells to promote its infectivity [1]. Moreover, *Salmonella*‐containing vacuole (SCV) can form within infected macrophages, which enables *Salmonella* to effectively evade the host immune response. Infected macrophages undergo a reprogramming of cellular oxidative metabolism, producing metabolic byproducts that serve as an energy source for *Salmonella* and as virulence activation signals, thus promoting further infection [2].

The host defends against *Salmonella* infection primarily through two mechanisms. The innate immune system, critical in the early stages of *Salmonella* infection, involves various cells playing distinct roles. For example, neutrophils promote extensive interferon‐gamma (IFN‐γ) release during the acute early infection of *Salmonella*, which effectively prevents extensive *Salmonella* replication and mitigating further infection (Figure 1). The adaptive immune response also plays a significant role in *Salmonella* clearance. CD4^+^ T cells predominantly drive this response, while the absence of B cells or CD8^+^ T cells does not seem to significantly affect *Salmonella* clearance.

**Figure 1:**
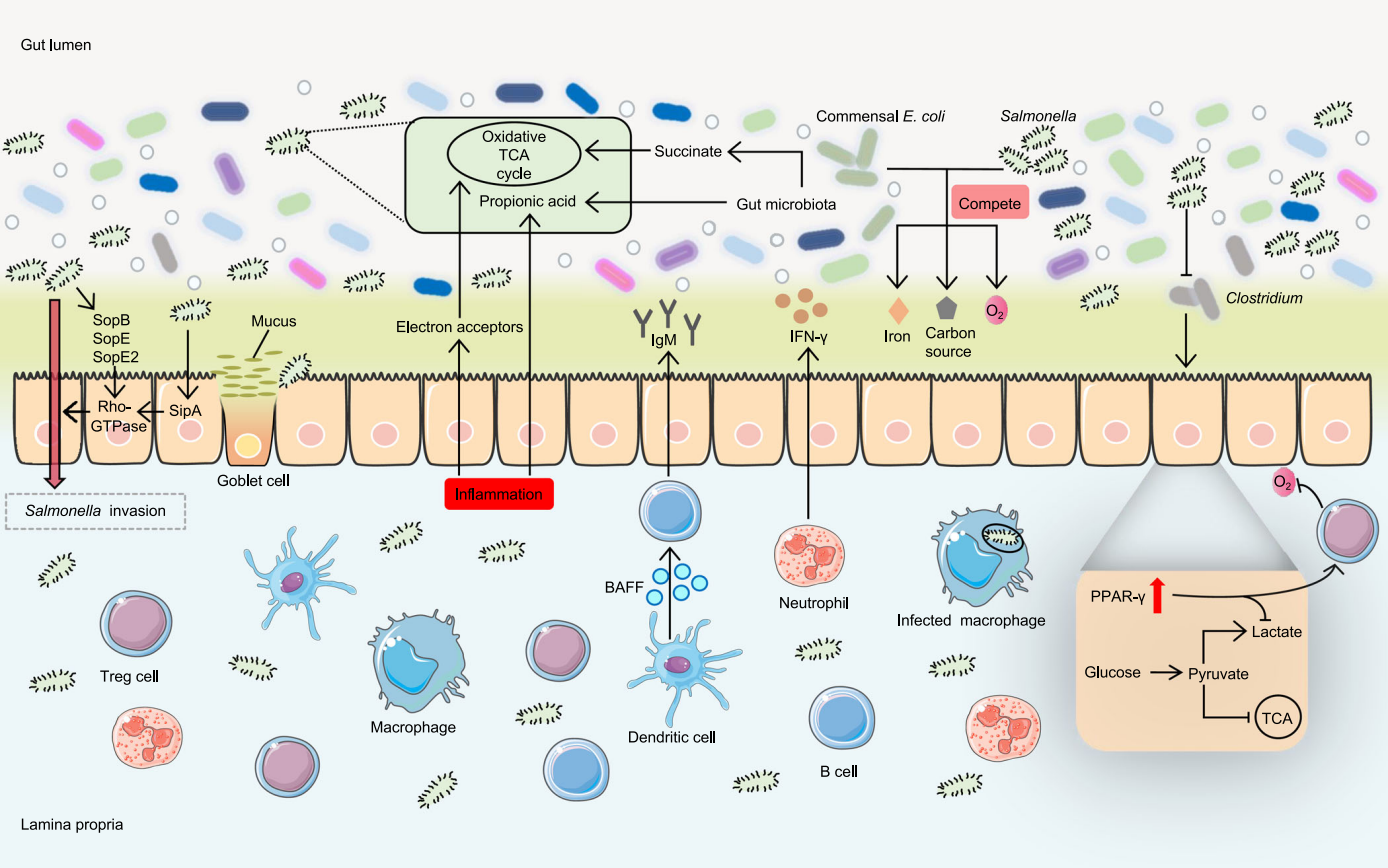
*Salmonella* invasion and interaction with gut microbiota. *Salmonella* utilizes its virulence factors SopB, SopE, SopE2, and SipA to activate the Rho‐GTPase signaling in intestinal epithelial cells and trigger invasion. After invasion, such as neutrophil secretory interferon‐gamma (IFN‐γ), dendritic cell can secrete B cell‐activating factor (BAFF) to promote B cells to secrete immunoglobulin M (IgM) antibody. In addition, the electron receptors produced by inflammation help *Salmonella* use microbial‐derived fermentation product succinate to promote its complete oxidation by the tricarboxylic acid (TCA) cycle in host. Inflammation also helps *Salmonella* use microbial‐derived fermentation product propionic acid. Furthermore, *Salmonella* competes with gut microbes, such as commensal *E. coli*, for nutrients, such as iron, carbon sources, and oxygen. *Salmonella* can also mediate host cell metabolism by eliminating *Clostridium*, thereby causing a shift in host cells from oxidative metabolism to lactic acid fermentation, which leads to increased epithelial cell oxygenation. However, when activated, host peroxisome proliferator‐activated receptor gamma (PPAR‐γ) signaling reduces lactate production and interacts with regulatory T cells to maintain hypoxia in the intestine. *E. coli*, *Escherichia coli*.

*Salmonella* interacts with the host–microbiota through diverse mechanisms, including competing for nutrients, influencing host cell metabolism. This review delves into how *Salmonella* promotes its expansion and replication within the host through immunity and metabolism, provides a comprehensive overview of this pathogenic mechanism in relation to the host and gut microbiota, furthering our understanding of *Salmonella* infection.

## 1 *SALMONELLA* INVASION AND INFECTION

*Salmonella* invades the host by utilizing the *Salmonella* pathogenicity island (SPI) encoding the type III secretion system (T3SS). The SPI‐1 encoded T3SS‐1 transfers bacterial effector proteins into host intestinal epithelial cells and induces cytosis. T3SS effectors such as SopE, SopE2, SopB, and SipA can disrupt the tight junctions between intestinal epithelial cells by acting as Rho‐GTPase agonists (Figure 1). Once invading the epithelium, *Salmonella* replicates in the cytoplasm to promote its expansion, and cause intestinal inflammation, which promotes *Salmonella* infection in host. On the one hand, inflammation can stimulate the release of compounds, like, tetrathionate and nitrates, which provide growth advantages as respiratory electron receptors, and encourage its utilization of derivatives, such as succinate [1] (Figure 1). On the other hand, inflammation disrupts the gut microbial balance and helps *Salmonella* to utilize various fermentation products derived from the gut microbiota, such as propionic acid [3]. In addition, *Salmonella* can also use immune evasion mechanisms to promote its infection. *Salmonella* can replicate within SCVs after endocytosis by macrophages and further inducing SCV acidification, by utilizing the CadC‐YdiV axis, halting flagellar synthesis to evade host immunity [4]. Additionally, *Salmonella* also has a unique immune evasion mechanism against host autophagic defenses through SPI‐1 effector SopB to mediate autophagy evasion [5]. In conclusion, *Salmonella* exhibits a unique mechanism to host‐induced inflammation and immune defense, which lays the foundation for systemic infection.

## 2 HOST CELL METABOLIC REPROGRAMMING

**Figure 2:**
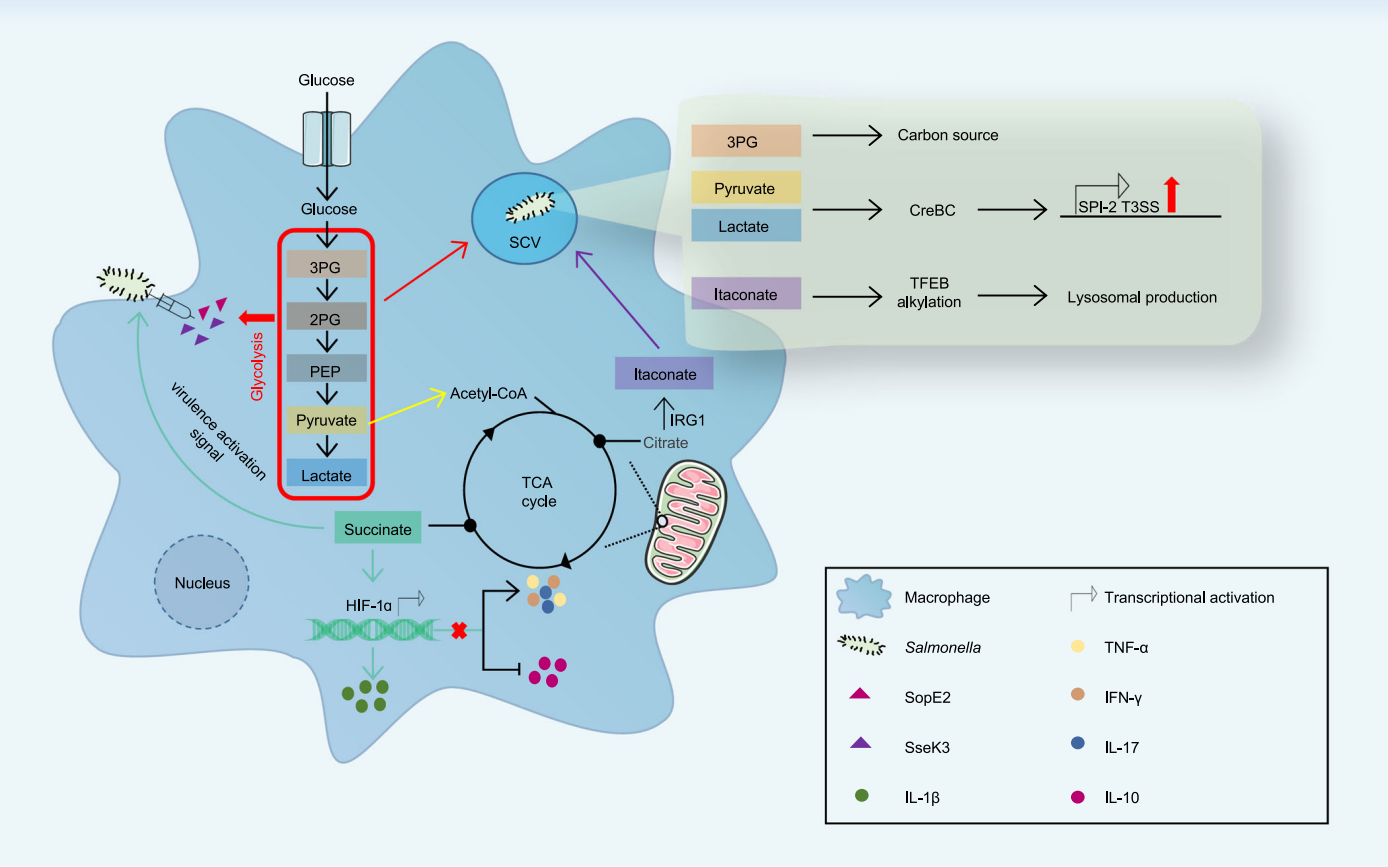
Host cell metabolism reprogramming. *Salmonella* infection induces metabolic reprogramming of macrophages, using its virulence factor effectors SopE2 and secreted effector K3 (SseK3) to enhance the glycolytic process, where metabolites such as 3‐phosphoglycerate (3PG) can be used by *Salmonella* as a carbon source for replication, and pyruvate and lactate can be sensed by the *Salmonella* two‐component system carbon source responsive (CreBC) to activate the *Salmonella* pathogenicity island‐2 (SPI‐2) expression. At the same time, the tricarboxylic acid (TCA) cycle is inhibited in macrophages, where increased levels of the metabolic intermediate, itaconate, can induce lysosome production through the transcription factor EB (TFEB) alkylation and produce a scavenging effect on bacteria. In addition, the metabolic intermediate succinate inhibits *Salmonella* by activating hypoxia‐inducible factor‐1 alpha (HIF‐1α) to release interleukin‐1β (IL‐1β), but *Salmonella* can also use the accumulated succinate in the host as a virulence activation signal to defend against host immunity. Blocking HIF‐1α expression can promote the secretion of the proinflammatory cytokines interferon‐gamma (IFN‐γ), tumor necrosis factor‐alpha (TNF‐α), and IL‐17 and inhibit IL‐10 secretion, which in turn is beneficial to *Salmonella*. PEP, phosphoenolpyruvate; SCV, Salmonella‐containing vacuole.

During systemic infection, infected macrophages exhibit the Warburg effect, characterized by increased glycolysis and suppression of tricarboxylic acid (TCA) cycle. The upregulation of glycolytic processes leads to the accumulation of glycolytic intermediates, such as 3‐phosphoglycerate (3PG), pyruvates, and lactates. 3PG can serve as a carbon source for intracellular replication of *Salmonella*, rescuing the bacteria from low glucose conditions. While pyruvates and lactates via the macrophage‐sensing two‐component system carbon source responsive (CreBC) to activate the *SPI‐2* gene [2] (Figure 2). Previous studies have shown that *Salmonella* Typhimurium secreted effector K3, an SPI‐2 effector, further contributes to the upregulation of glycolysis, induction of macrophage apoptosis, and concurrently suppresses the expression of inflammatory factors [6] (Figure 2). Notably, the upregulation of glycolysis was previously thought to be a protective effect induced by the host, through which adenosine triphosphate can be obtained more quickly to support biosynthesis and meet the metabolic demands of cell proliferation. However, studies on human macrophages have shown that inhibiting glycolysis within host cells can reduce the growth of intracellular *Salmonella*, which is related to increasing reactive oxygen species production [7]. In addition, the accumulated lactate is produced by glycolysis in both mouse and human macrophages, it appears to be only required for *Salmonella* SPI‐2 in mice [8]. The specific role of lactate in human macrophages during *Salmonella* infection remains unclear, warranting further investigation.

Inhibition of the TCA cycle reduces the levels of key intermediates like citrate, conversely, the levels of succinate, itaconate increase in response to *Salmonella* infection [9]. These metabolic changes are pathways by which host cells react to infection. *Salmonella* infection can decrease host citrate levels, potentially due to the transfer of citrate from the TCA cycle to lipid biosynthesis. Elevated itaconate induces alkylation of the transcription factor EB alkylation to induce lysosomal generation, aiding bacterial clearance [10] (Figure 2). Succinate can upregulate hypoxia‐inducible factor‐1 alpha (HIF‐1α) expression, ultimately leading to increased interleukin‐1β production. In addition, HIF‐1α expression inhibition decreases *Salmonella* viability, potentially through the epidermal growth factor receptor–HIF‐1α axis, since inhibiting this pathway can enhance proinflammatory cytokine IFN‐γ, tumor necrosis factor‐alpha, interleukin‐17 (IL‐17) production, while reducing anti‐inflammatory IL‐10 secretion [11] (Figure 2). Overall, *Salmonella* can reprogram infected macrophage glucose metabolism, inducing a sufficient accumulation of macrophage‐derived carbon sources and signals, which were utilized as an energy source to support intracellular replication and virulence of *Salmonella*.

## 3 HOST IMMUNE RESPONSE TO *SALMONELLA*

When *Salmonella* infects the gut, the intestinal epithelium rapidly recognizes the pathogen through the neuronal apoptosis inhibitory protein/nod‐like receptors‐family CARD‐containing protein 4 inflammasome. This recognition triggers a contraction of the epithelial layer, helpful in maintaining the epithelial barrier integrity and preventing further *Salmonella* invasion [12]. Macrophages can also clear *Salmonella* through extracellular traps release, referred to as ETosis [13]. Dendritic cells also produce B‐cell activating factor to stimulate the expansion of mature B cells and *Salmonella*‐specific immunoglobulin M production, which is necessary to prevent *Salmonella* infection [14] (Figure 1).

The adaptive immune response to *Salmonella* infection involves CD4^+^ and CD8^+^ T‐cell activation. CD4^+^ T cells play a key role in persistent *Salmonella* infection by producing IFN‐γ to inhibit *Salmonella* reactivation in the intestine. Interestingly, the protective effects of CD4^+^ T cell may exhibit heterogeneity in different organs. In chronically infected mice, research has found that compared to the spleen and lymph nodes, the liver CD4^+^ T cells can also generate IL‐10, hindering *Salmonella* clearance [15]. During *Salmonella* infection, CD8^+^ T cells have been shown to exhibit delayed expansion and contraction, while play a protective role in primary infection after administration of a live attenuated *Salmonella* vaccine.

## 4 *SALMONELLA*—MICROBIOTA INTERACTIONS

### Competing for nutrients

*Escherichia coli* can reduce *Salmonella* intestinal colonization by competing for iron, and carbon sources (Figure 1). Reduced carbon sources can induce caloric restriction, which has previously been shown to reduce *Salmonella* infection through nitric oxide (NO). *Salmonella* employs siderophores to compete for iron from the host. Blocking siderophores effectively reduce *Salmonella* colonization in the gut. Recent studies have shown that *Bacteroides thetaiotaomicron* secreted siderophore‐binding lipoprotein XusB to utilizing siderophores, but the XusB‐bound enterobactin are utilized by *Salmonella*, allowing pathogenic bacteria to evade host nutritional immunity [16]. *E. coli* can also compete with *Salmonella* for oxygen to produce colonization resistance to *Salmonella* [17] (Figure 1). *E. coli* acquires nitrate from epithelial sources, but *Salmonella* is limited by the chemotactic receptors methyl‐accepting chemotaxis proteins, which prevent it from accessing the *E. coli* niche to obtain epithelial‐derived nitrate. Meanwhile, commensal *E. coli* can enter *Salmonella*'s niche to compete for macrophage‐derived nitrate, thereby enhance resistance against *Salmonella* colonization [18].

### Affecting cellular metabolism

*Salmonella* uses virulence factors to eliminate butyric acid‐producing *Clostridium* from the gut microbiota, leading to increased epithelial oxygenation (Figure 1). This, in turn, drives the cytochrome bd II oxidase‐dependent expansion of *Salmonella* within the intestinal lumen through aerobic and nitrate respiration [19]. *Salmonella* can facilitate its own colonization and infection by disrupting gut microbial homeostasis and promoting oxidative metabolism. The agonists of peroxisome proliferator‐activated receptor gamma can decrease the production of lactate by host cells and synergize with regulatory T cells to maintain colonic hypoxia, which contributes to the production of short‐chain fatty acids by gut microbes and helps maintain intestinal homeostasis [20] (Figure 1). This mechanism represents a potential metabolic pathway target for treating *Salmonella* infections.

## 5 INSIGHTS AND FUTURE DIRECTIONS

During *Salmonella* infection, the production of inflammation can initially serve as a protective mechanism for the host, but certain byproducts of the inflammatory response may facilitate the movement of *Salmonella* in vivo, which can contribute to the survival and virulence of *Salmonella*. While the host mounts an immune response against *Salmonella* infection, the bacteria possess their own mechanisms to evade or circumvent the host defenses. *Salmonella* infection offers valuable insights into how the bacterium manipulates host metabolism to favor its own proliferation within the host. Yet, additional research is needed to fully elucidate the molecular mechanisms involved.

*Salmonella* infection can disrupt the balance of gut commensal bacteria by competing for nutrients, changing the structure of the microbial community, and using microbial metabolites to fuel its own energy metabolism. The current research sheds light on the intricate interactions between *Salmonella*, the host, and gut microbiota in terms of immunity and metabolism; however, further research is needed to fully elucidate the immune and metabolic mechanisms underlying these interactions. By gaining a better understanding of these processes, it has the potential to harness the host's metabolic regulation to enhance immunity and provide better defense against pathogenic bacterial infections.

## AUTHOR CONTRIBUTIONS

**Bingxin Tang**: Writing—original draft; writing—review and editing; conceptualization. **Wenwen Cui**: Writing—review and editing. **Xiao Li**: Writing—review and editing. **Huan Yang**: Conceptualization; writing—review and editing; funding acquisition.

## CONFLICT OF INTEREST STATEMENT

The authors declare no conflict of interest.

## ETHICS STATEMENT

No animals or humans were involved in this study.

## Data Availability

This manuscript does not generate any code or data. Supporting Information materials (graphical abstract, slides, videos, Chinese translated version, and update materials) may be found in the online DOI or iMeta Science http://www.imeta.science/imetaomics/.
